# Cardiac Autonomic Dysfunction in Type 2 Diabetes – Effect of Hyperglycemia and Disease Duration

**DOI:** 10.3389/fendo.2014.00130

**Published:** 2014-08-08

**Authors:** Mika P. Tarvainen, Tomi P. Laitinen, Jukka A. Lipponen, David J. Cornforth, Herbert F. Jelinek

**Affiliations:** ^1^Department of Applied Physics, University of Eastern Finland, Kuopio, Finland; ^2^Department of Clinical Physiology and Nuclear Medicine, Kuopio University Hospital, Kuopio, Finland; ^3^School of Design, Communication and IT, University of Newcastle, Newcastle, NSW, Australia; ^4^School of Community Health, Centre for Research in Complex Systems, Charles Sturt University, Albury, NSW, Australia

**Keywords:** heart rate variability, hyperglycemia, blood glucose, HbA1c, duration, cardiac autonomic neuropathy

## Abstract

Heart rate variability (HRV) is reduced in diabetes mellitus (DM) patients, suggesting dysfunction of cardiac autonomic regulation and an increased risk for cardiac events. The aim of this paper was to examine the associations of blood glucose level (BGL), glycated hemoglobin (HbA1c), and duration of diabetes with cardiac autonomic regulation assessed by HRV analysis. Resting electrocardiogram (ECG), recorded over 20 min in supine position, and clinical measurements of 189 healthy controls and 93 type 2 DM (T2DM) patients were analyzed. HRV was assessed using several time-domain, frequency-domain, and non-linear methods. HRV parameters showed a clear difference between healthy controls and T2DM patients. Hyperglycemia was associated with increase in mean heart rate and decrease in HRV, indicated by negative correlations of BGL and HbA1c with mean RR interval and most of the HRV parameters. Duration of diabetes was strongly associated with decrease in HRV, the most significant decrease in HRV was found within the first 5–10 years of the disease. In conclusion, elevated blood glucose levels have an unfavorable effect on cardiac autonomic function and this effect is pronounced in long-term T2DM patients. The most significant decrease in HRV related to diabetes and thus presence of autonomic neuropathy was observed within the first 5–10 years of disease progression.

## 1 Introduction

Heart rate variability (HRV) is a commonly used tool to assess the functioning of cardiac autonomic regulation. The autonomic nervous system (ANS) regulates heart rate (HR) through sympathetic and parasympathetic (vagal) branches where the sympathetic activity increases HR and decreases HRV, whereas parasympathetic activity decreases HR and increases HRV (1). Two apparent components of HRV are the low frequency (LF, ranging from 0.04–0.15 Hz) component mediated by both sympathetic and parasympathetic nervous activities and the high frequency (HF, 0.15–0.4 Hz) component mediated almost solely by parasympathetic nervous activity (1, 2).

Heart rate variability is reduced in diabetes mellitus (DM) patients, suggesting dysfunction of cardiac autonomic regulation, which has been previously shown to be associated with increased risk for adverse cardiac events (3). Cardiac autonomic neuropathy (CAN), which results from damage to autonomic nerve fibers that innervate the heart and blood vessels, is a serious complication of DM (4). During progression of CAN, the parasympathetic nerve fibers innervating the heart are affected before the sympathetic nerve fibers leading to a reduced heart rate variability (5). Reduced HRV is recognized as an early indicator of CAN, but reduction of HRV has been observed also in patients without evidence of CAN when using traditional tests such as the Ewing battery (2, 6, 7). Standard time and frequency-domain analysis of HRV combined with cardiovascular autonomic reflex tests are used in clinical assessment of CAN (8), but recently several non-linear methods for assessing CAN have been proposed (9, 10).

The pathogenesis of diabetic neuropathies is complex, but long-lasting hyperglycemia is responsible for chronic metabolic perturbations (mainly increased activation of the polyol pathway and increased production of harmful metabolites) leading to neuronal damage (7, 11–13). Therefore, the blood glucose targets suggested for most patients with diabetes are fasting blood glucose level (BGL) 7 mmol/L and glycated hemoglobin (HbA1c) 7% (53 mmol/mol), but it is recommended that these blood glucose targets should be individualized to meet patient needs (14).

Several studies on the association between BGL and HRV have been conducted in order to better understand the autonomic dysfunction related to diabetes, both with and without CAN. In general, reduced HRV has been observed in diabetic patients (15–17). In Ref. (16), the HF component of HRV was reduced in subjects with DM. However, in Ref. (17), reduction of the LF component of HRV was observed in diabetics as well as in subjects with impaired fasting BGL. Furthermore, HRV has been shown to be inversely associated with BGL, indicated by negative correlations between both LF and HF component powers and BGL (17). In addition, an increased LF/HF power ratio has been shown during hyperglycemia in controls and diabetics without CAN (18).

Based on these studies, it has been suggested that cardiac autonomic dysfunction is associated with impaired glucose tolerance (IGT) tested with an oral glucose tolerance test, but not with impaired fasting glucose (7, 19). Nevertheless, effective glycemic control plays a central role in reducing the risk of CAN, but the associations between BGL and autonomic dysfunction need to be further studied. Also, the timeline of these changes in ANS function should be investigated further to better understand the progression of CAN. In addition, the associations between autonomic dysfunction and other risk factors, such as obesity, hypertension, or increased cholesterol levels, need to be considered in future studies.

The aim of the present study was to examine the associations between hyperglycemia and ANS dysfunction in patients with type 2 DM (T2DM). ANS function was assessed by using a wide range of time-domain, frequency-domain, and non-linear HRV analysis methods. Glycemic control was determined by fasting BGL and HbA1c measurements. HbA1c is commonly used to identify average plasma glucose concentration over 3 months prior to the measurement. In addition, other risk factors such as body weight, blood cholesterol, and blood pressure were also considered. Furthermore, association of the disease duration with ANS dysfunction in diabetes was examined to reveal the timeline of changes in cardiac autonomic regulation.

## 2 Materials and Methods

### 2.1 Subjects and recordings

189 healthy controls and 93 type 2 diabetes mellitus patients who were participants of a health screening clinic at Charles Sturt University were included in the study. Some of the subjects were measured more than once (1–5 visits per subject during 2002–2012, on average two visits per each diabetic subject) resulting in a total of 273 control and 199 T2DM measurements. Subjects with history or clinical evidence of heart failure, atrial fibrillation, or myocardial infarction were excluded from the study. None of the diabetic patients showed clinical evidence of CAN using the Ewing battery of tests (20).

Fasting blood samples were taken to measure blood glucose, glycated hemoglobin, and blood cholesterol. BGL was measured clinically using an Accu-Chek Advantage II glucometer (Roche Australia P/L). Fasting plasma HbA1c, total cholesterol (TC), triglycerides (TG), and high-density lipoprotein cholesterol (HDL) were measured by standard techniques. TC and TG were determined with a commercial enzymatic kit. HDL was determined by immunoinhibition assay. Low-density lipoprotein cholesterol (LDL) was calculated according to the Friedewald formula. Systolic and diastolic blood pressure (SBP and DBP) were measured in a supine position using a Welch–Allyn blood pressure recorder. Use of anti-hypertensive drugs (ACEI, ARB, ${\hat{\text{l}}}^{2}$-blockers), antidepressants (SNARI, SSRI, Amitriptyline hydrochloride), or anti-cholesterol drugs (statins) were identified. The clinical characteristics of the healthy controls and T2DM patients who participated in the study are summarized on Table 1.

**Table 1:** **Clinical characteristics of the healthy control subjects and T2DM patients participating the study**.

Variable	Units	Healthy controls (*N* = 273)		T2DM patients (*N* = 199)		*p*^a^
		Median (50% CI)	*n* (males, %)	Median (50% CI)	*n* (males, %)	
Age	Years	62 (54–70)	273 (38)	66 (59–71)	199 (44)	**
Duration of diabetes	Years	–	–	8 (4–12)	199 (44)	–
BMI	kg/m^2^	26.5 (23.9–29.9)	268 (38)	29.5 (26.2–33.7)	191 (45)	***
BGL	mmol/l	4.8 (4.3–5.3)	267 (38)	7.2 (5.7–10.2)	189 (44)	***
HbA1c	%	5.6 (5.4–5.8)	77 (36)	6.8 (6.2–7.6)	121 (43)	***
	mmol/mol	38 (36–40)	77 (36)	51 (44–60)	121 (43)	***
Total cholesterol (TC)	mmol/l	5.1 (4.6–5.7)	178 (36)	4.4 (3.5–5.3)	118 (47)	***
LDL	mmol/l	3.1 (2.6–3.5)	115 (34)	2.4 (1.6–3.1)	103 (48)	***
HDL	mmol/l	1.4 (1.1–1.7)	172 (35)	1.2 (1.0–1.4)	115 (49)	***
Triglyceride	mmol/l	1.0 (0.8–1.6)	127 (35)	1.6 (1.1–2.2)	112 (47)	***
TC/HDL	–	3.6 (3.0–4.6)	172 (35)	3.6 (3.0–4.2)	115 (49)	N.S.
SBP	mmHg	126 (118–140)	269 (38)	132 (125–145)	198 (44)	***
DBP	mmHg	79 (70–83)	269 (38)	79 (71–84)	198 (44)	N.S.
Medication^b^
Anti-hypertensive			63 (30)		139 (40)	***
Antidepressant			17 (18)		19 (32)	N.S.
Anti-cholesterol			8 (25)		22 (68)	**

***p ≤ 0.0001; **p ≤ 0.001; *p ≤ 0.027.

*Values are expressed as Median (50% confidence interval)*.

*^a^Mann–Whitney U test for the difference between control subjects and T2DM patients*.

*^b^Number of subjects using one or more of the listed medication types (the difference between the groups evaluated using cross-tabulation and Chi-squared statistics)*.

A supine resting electrocardiogram (ECG) was recorded over 20 min at 400 Hz sampling rate using a lead II configuration (Maclab ADInstruments, Australia) for all participants. An adaptive QRS detector algorithm was applied to extract the beat-to-beat RR intervals from the ECG data. The very low frequency trend components (frequencies below 0.04 Hz) were removed from the RR interval time series by using a smoothness priors method (21). Furthermore, the non-equidistantly sampled RR series were interpolated (4 Hz cubic spline interpolation) to have evenly sampled data for spectral analysis. Respiratory frequency was estimated from the ECG R-wave amplitude changes and utilized in HF component estimation.

The study was approved by the Charles Sturt University Human Ethics Committee and written informed consent was obtained from all participants.

### 2.2 Heart rate variability analysis

HRV was assessed using several time-domain, frequency-domain, and non-linear analysis parameters, by following the guidelines given in Ref. (2). The frequency-domain parameters were computed from the detrended and interpolated (equidistantly sampled) RR interval series, whereas time-domain and non-linear parameters were computed from the detrended but not interpolated RR series.

The time-domain parameters included the mean RR interval, standard deviation of normal-to-normal RR intervals (SDNN), root mean square of successive RR interval differences (RMSSD), and percentage of successive RR intervals with difference bigger than 50 ms (pNN50). Two geometric measures computed from the RR interval histogram were also considered. These were the HRV triangular index (HRVi), which gives the total number of RR intervals within the time series (integral of the histogram) divided by the number of RR intervals at the modal bin of the histogram, and the triangular interpolation of RR interval histogram (TINN), which is the baseline width of a triangle fitted to the histogram.

The frequency-domain parameters included LF and HF component powers and total spectral power. LF and HF powers were also computed in normalized units, which were obtained by dividing the absolute powers with total spectral power and multiplying by 100 to give values as a percentage. The frequency-domain parameters were extracted from the RR interval spectrum, which was estimated using autoregressive (AR) spectrum estimation with model order 20. The advantage of AR spectrum is that the spectrum estimate can be decomposed into distinct components using spectral factorization (22). The LF and HF component powers were computed from the factorized spectrum by summing the spectral components centered within 0.04–0.15 and 0.15–0.5 Hz, respectively. The upper limit of HF band was increased to 0.5 Hz because the respiratory frequency of some subjects was over 0.4 Hz. Subjects with respiratory frequency below 0.15 Hz was excluded.

Furthermore, four commonly used non-linear HRV methods were used to assess HRV. The Poincaré plot is a scatter plot between successive RR intervals and provides indexes for short-term variability (SD1) and long-term variability (SD2), where both SD1 and SD2 are non-linearly connected to time-domain parameters (23). Sample entropy (SampEn) is a commonly used measure of signal complexity and was computed using an embedding dimension of *m* = 2 and tolerance of 0.2 times the standard deviation of the time series (24). Correlation dimension is also a measure of signal complexity and is expected to carry information of the minimum number of dynamic variables needed to model the underlying system (25). Finally, detrended fluctuation analysis (DFA) is a measure of self-affinity of a signal, separated here into short-term (α_1_, 4–16 beat fluctuations), and long-term (α_2_, 16–64 beat fluctuations) fluctuations within the RR data (26).

### 2.3 Statistical analysis

Non-parametric tests were used because some of the HRV parameters were not normally distributed. Data are presented as median values (50% confidence interval). The Mann–Whitney U test was used to test the group differences between healthy control subjects and T2DM patients as well as for testing differences between T2DM patients grouped according to disease duration, measured blood glucose, or HbA1c. These group differences were tested mainly to discover the most significant differences in HRV parameters between the groups, but also the clinical characteristics were tested to observe differences between healthy controls and T2DM patients. In case of categorical variables (i.e., medication use), cross-tabulation and Chi-squared statistics were used to test the difference between the groups. Furthermore, Spearman’s rank correlation coefficient was applied for measuring statistical dependence between HRV parameters and disease duration, BGL, and HbA1c within the diabetes group. Finally, stepwise linear regression analysis was applied to determine whether there were independent associations between HRV parameters and BGL, HbA1c, and disease duration. When evaluating the associations of HRV with BGL and HbA1c, the influence of disease duration, and medication use were taken into account by adding them as descriptive parameters in the regression model.

The significance level was set to α = 0.05 and corrected for multiple comparisons with false discovery rate (FDR) to yield a threshold of *p* ≤ 0.027.

## 3 Results

The clinical characteristics of the measured subjects are summarized in Table 1. The diabetes group was on average 4 years older and with a higher BMI compared to healthy control subjects. Within the T2DM patients, BGL varied between 3.3 and 20.1 mmol/L and HbA1c between 5.2 and 13.0% (33–119 mmol/mol), both of these indexes being significantly higher for diabetes group compared to controls. The diabetes group also had lower TC, LDL cholesterol, and HDL cholesterol, but higher triglycerides, compared to controls. Furthermore, the diabetes group had higher systolic blood pressure and the use of anti-hypertensive and anti-cholesterol medications were more common within the diabetes group compared to controls.

**Figure 1:**
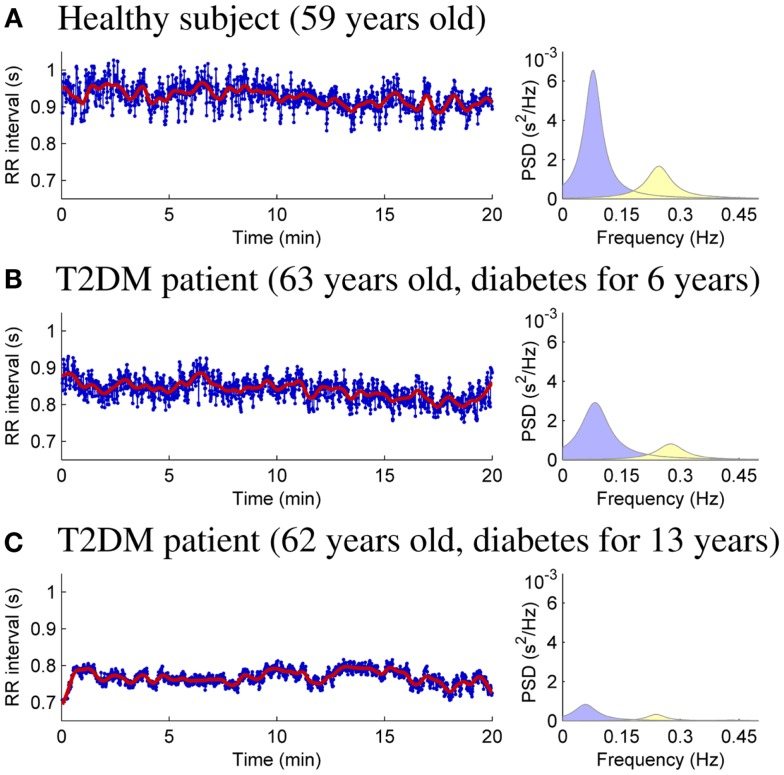
**RR interval time series (bold line showing the removed trend) and AR spectrum estimates for healthy control subject (A), T2DM patient diagnosed 6 years prior to measurement (B), and T2DM patient diagnosed 13 years prior to measurement (C)**.

HRV analysis of the 20-min resting measurements was then performed as described in Section 2. RR interval data and power spectral estimates for three representative subjects are shown in Figure 1. The very low frequency trend components removed from the RR interval series prior to analysis are illustrated over the RR interval data. The mean RR interval length and the powers of the LF and HF components are highest for the healthy control (Figure 1A) and lowest for theT2DM patient with longer disease duration for similar age (Figure 1C).

HRV analysis results are summarized in Table 2, which presents the median (50% confidence interval) values of each HRV parameter for healthy controls and T2DM patients. Multiple linear regression was applied to adjust the HRV parameter values for the confounding effects of gender, age, and BMI. Most of the HRV parameters showed a clear difference between control and diabetic subjects. It is observed that HR is increased (mean RR decreased) and HRV decreased in T2DM patients when compared to controls. However, normalized LF and HF powers did not show significant difference between the diabetes group and healthy controls.

**Table 2:** **HRV parameter values for healthy control subjects and T2DM patients and their correlations with duration of diabetes, BGL, and HbA1c within T2DM patients**.

HRV parameter (units)	Healthy controls vs. T2DM patients			Correlation coefficients^b^		
	Control (*N* = 273) Median (50% CI)	T2DM (*N* = 199) Median (50% CI)	*p*^a^	BGL (mmol/l)	HbA1c (%)	Duration (years)
Mean RR (ms)	963 (891–1051)	903 (804–1015)	***	−0.246**	−0.195*	−0.166*
SDNN (ms)	25.1 (19.0–31.9)	20.3 (14.2–27.7)	***	−0.218*	−0.253*	−0.383***
RMSSD (ms)	25.0 (18.1–33.1)	19.1 (12.5–28.0)	***	−0.229*	−0.250*	−0.346***
pNN50 (%)	3.56 (1.11–10.27)	2.02 (0.42–5.18)	**	−0.247**	−0.238*	−0.267**
HRVi	6.71 (5.29–8.49)	5.81 (3.99–7.22)	***	−0.238**	−0.230*	−0.398***
TINN (ms)	157 (119–214)	124 (91–171)	***	−0.149	−0.250*	−0.363***
LF power (dB)	25.4 (22.6–27.9)	23.1 (19.8–26.2)	***	−0.186*	−0.234*	−0.372***
HF power (dB)	22.5 (19.7–25.8)	21.2 (16.6–24.6)	**	−0.173*	−0.204*	−0.359***
Total power (dB)	27.9 (25.6–30.1)	25.9 (22.8–28.9)	***	−0.197*	−0.251*	−0.418***
LF power (n.u.)	62.0 (49.5–73.7)	62.3 (47.8–74.2)	N.S.	0.013	−0.053	−0.003
HF power (n.u.)	37.5 (25.7–50.2)	35.7 (25.1–51.4)	N.S.	−0.040	0.036	−0.034
SD1 (ms)	17.7 (12.8–23.4)	13.5 (8.9–19.8)	***	−0.229*	−0.250*	−0.346***
SD2 (ms)	30.6 (23.0–39.4)	25.0 (18.1–33.7)	***	−0.208*	−0.252*	−0.385***
SampEn	1.73 (1.55–1.85)	1.75 (1.62–1.88)	N.S.	−0.161*	0.227*	0.073
DFA, α_1_	1.00 (0.82–1.15)	1.01 (0.77–1.15)	N.S.	0.060	−0.056	0.013
DFA, α_2_	0.35 (0.29–0.43)	0.41 (0.31–0.50)	***	0.205*	0.187	0.193*
D2	0.47 (0.16–1.02)	0.27 (0.06–0.57)	**	−0.230*	−0.209*	−0.263**

***p ≤ 0.0001; **p ≤ 0.001; *p ≤ 0.027.

*HRV parameter values are expressed as Median (50% confidence interval), adjusted for age, gender and BMI*.

*^a^Mann–Whitney *U* test for the difference between control subjects and T2DM patients*.

*^b^Spearman’s rank correlation coefficients for the statistical dependence between HRV parameters and BGL, HbA1c and disease duration (computed within the diabetes group)*.

The effect of glycemia and disease duration on different HRV parameters were then evaluated within the data of T2DM patients by computing Spearman’s rank correlation coefficients between each HRV parameter and BGL, HbA1c, and disease duration. Spearman’s correlations are presented in Table 2. Mean RR interval and most of the HRV parameters were negatively correlated with both BGL and HbA1c. However, DFA α_2_ showed a significant positive correlation with BGL and sample entropy a positive correlation with HbA1c. Most of the HRV parameters showed also a strong negative correlation with disease duration, whereas mean RR showed only a small negative correlation with disease duration.

**Figure 2:**
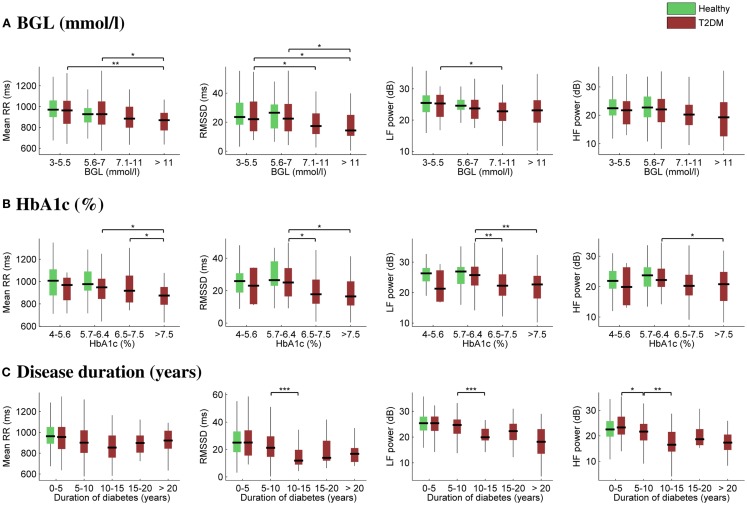
**Box plots of selected HRV associations with BGL (A), HbA1c (B), and duration of diabetes (C)**. On each box, the central mark is the median, the edges of the box are the 25th and 75th percentiles, and the whiskers extend to the most extreme parameter values excluding outliers. Significant differences between all the “boxes” **(A,B)** or between successive “boxes” **(C)** were tested using the Mann–Whitney U test (***p ≤ 0.001; **p ≤ 0.01; *p ≤ 0.05).

The effect of glycemia on selected HRV parameters is further illustrated in Figures 2A,B. In order to observe HRV changes related to glycemia, the diabetes group was divided into four subgroups according to BGL (3–5.5, 5.6–7, 7.1–11, and >11 mmol/L) and HbA1c (4–5.6, 5.7–6.4, 6.5–7.5, and >7.5%). The number of measurements within each subgroup varied between 32 and 66, except for the lowest HbA1c subgroup, which consisted of only four measurements. The healthy subjects were divided only into the first two glycemic (3–5.5 and 5.6–7 mmol/L) subgroups. No remarkable changes were observed between the first two glycemic (control or diabetes) subgroups, i.e., when BGL is below 7 mmol/L and HbA1c below 6.4% (46 mmol/mol), but mean RR interval and HRV were decreased for the two highest glycemic subgroups for diabetic patients.

Correspondingly, the effect of disease duration on HRV is illustrated in Figure 2C. The diabetes group was divided into five subgroups according to the time after the diagnosis. The number of measurements within each subgroup varied between 17 and 66. Mean RR interval did not change significantly, whereas a substantial reduction in HRV was observed as a function of disease duration. Moreover, the decrease in HRV took place mainly during the first 5–10 years of the disease.

**Figure 3:**
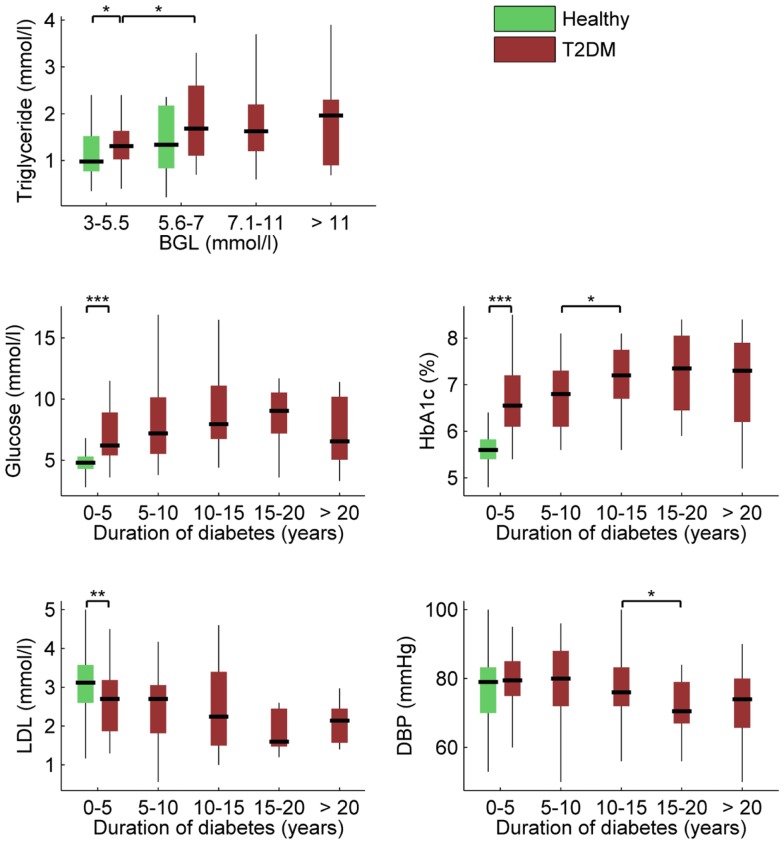
Box plots of most significant associations between clinical characteristics, BGL, HbA1c, and duration of diabetes (***p ≤ 0.001; **p ≤ 0.01; *p ≤ 0.05). Descriptions as in Figure 2.

Associations between BGL, HbA1c, disease duration, and other clinical characteristics provided in Table 1 were next examined using Spearman’s correlation. BGL was naturally associated with HbA1c, but also with disease duration (*r* = 0.185, *p* = 0.011), triglyceride level (*r* = 0.201, *p* = 0.036), and use of anti-cholesterol medication (*r* = 0.145, *p* = 0.046). HbA1c was also associated with disease duration (*r* = 0.226, *p* = 0.013) but not with any other clinical measure except BGL. In addition, disease duration was associated with LDL (*r* = -0.239, *p* = 0.015) and DBP (*r* = −0.187, *p* = 0.008). These main associations within the clinical variables are illustrated in Figure 3.

Since glycemic values (BGL and HbA1c) were associated with disease duration and medication use, a stepwise linear regression was applied to systematically examine:

1. The independent associations of different HRV parameters with BGL, HbA1c, and disease duration.
2. The independent associations of HRV parameters with BGL and HbA1c after including disease duration and medication use in the model as descriptive parameters.

Among the HRV parameters, the best descriptive parameters for BGL were found to be mean RR, SampEn, and absolute LF power. When disease duration and medication use were included in the model as descriptive parameters, Mean RR, SampEn, and LF power remained as the three most significant descriptive parameters for BGL. Similarly, the best descriptive HRV parameter for HbA1c was total spectral power and the model accuracy was not significantly improved by adding any other HRV parameter. When disease duration and medication use were included in the model, disease duration came out as the sole significant descriptive parameter for HbA1c. Finally, the sole significant descriptive HRV parameter for disease duration was total spectral power, and when medication use was included in the model, total spectral power remained as the sole significant descriptive parameters.

**Figure 4:**
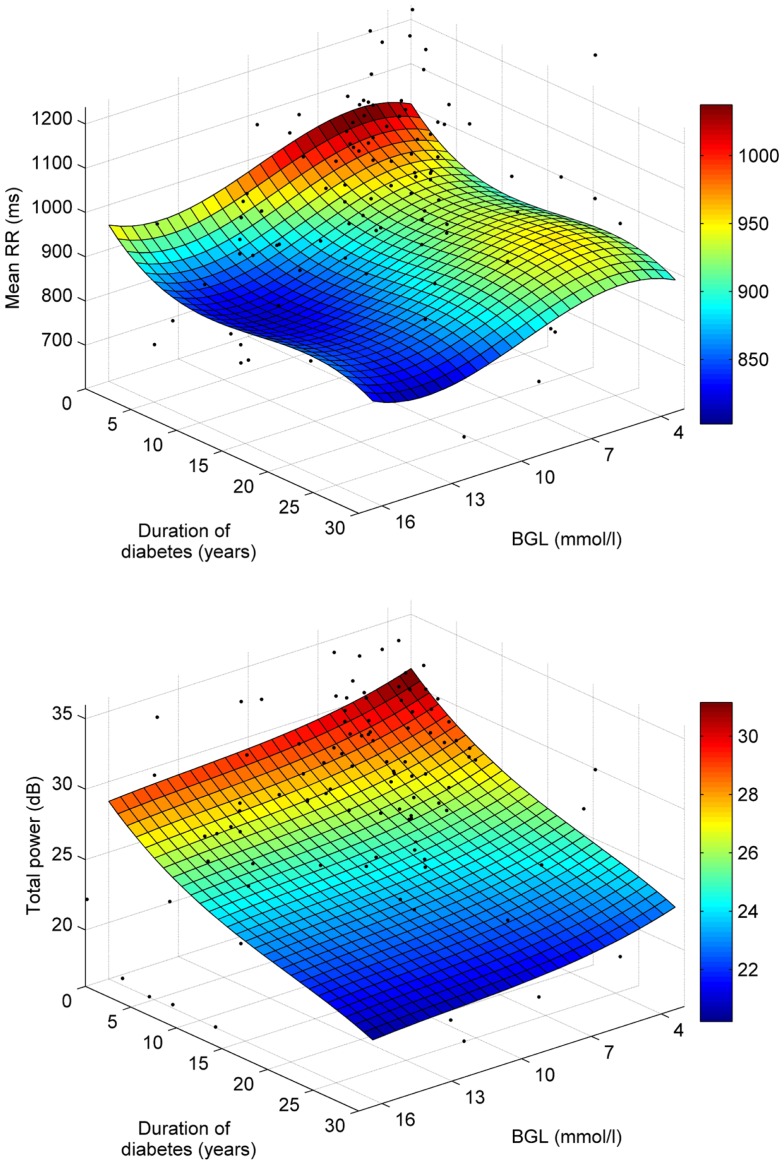
**Polynomial least squares surface fits for the associations of mean RR (top) and total power (bottom) with BGL and disease duration**.

The associations of mean RR and total spectral power with BGL and disease duration are further illustrated in Figure 4, which shows third order polynomial surface fits on the individual data points. Also from this Figure, it is observed that the decrease of mean RR is stronger along the BGL axis, whereas decrease in total power is more strongly associated with disease duration.

## 4 Discussion

In this paper, the associations of cardiac autonomic regulation assessed by HRV analysis with BGL, HbA1c, and duration of diabetes in T2DM patients were examined. In summary, HR was increased and HRV decreased in hyperglycemia. The mean RR interval decreased (HR increased) consistently as a function of BGL and HbA1c as shown by Table 2 and Figure 2. Most of the HRV parameters decreased as a function of BGL and HbA1c, but normalized powers of LF or HF components were not associated with glycemia. This suggests that cardiac autonomic regulation is reduced in hyperglycemia without significant change in sympatho-vagal balance.

In the stepwise regression, the most significant descriptors for BGL were Mean RR, SampEn, and LF power, whereas for HbA1c total spectral power was the sole significant descriptive parameter. The decrease of mean RR and LF power indicates that the reduction of HRV in hyperglycemia could be dominated by the sympathetic branch of the ANS in agreement with known physiology, where increased BGL leads to increase in sympathetic nervous activity (27). The LF component of HRV is also affected by baroreflex activity, which is known to be reduced in diabetes but its association with hyperglycemia is unknown.

In addition to linear measures, some of the non-linear measures of HRV were associated with BGL and HbA1c. The Poincaré plot indices SD1 and SD2 both decreased as a function of increased BGL and HbA1c, which is understandable considering the connections of these indices to time-domain measures of HRV (23). One interesting finding was that sample entropy was the second most significant descriptor (after mean RR) in the stepwise regression analysis for BGL, indicating that hyperglycemia induces changes in RR time series complexity, which is not detectable by standard linear methods. However, while SampEn correlated negatively with BGL, its correlation with HbA1c was positive. The long-term correlations estimated by DFA α_2_ were also found to be increased in hyperglycemia, but the reliability of this parameter is not optimal when computed from short-term recordings (26) and its values are overall lower due to detrending performed in this study. An alternative explanation is that acute effects such as raised BGL affect initially parasympathetic modulation, whereas chronic hyperglycemia becomes more pronounced with increased sympathetic modulation of the HR, which is reflected by the increased SampEn and DFA α_2_.

It should be mentioned that the HRV values observed for the lowest glycemic range may be affected by hypoglycemia, which is a common side effect of medication and associated with adverse cardiac outcomes possibly by affecting sympathetic nervous system function (13).

HRV was also strongly associated with the duration of diabetes. Mean RR interval, on the other hand, did not show consistent changes as a function of disease duration. HRV was decreased as a function of disease duration illustrated by significant negative correlations between most of the HRV parameters and disease duration (see Table 2). However in the stepwise regression analysis, the sole significant descriptive parameter for disease duration was the total spectral power. The appearance of total power as the most significant descriptive parameter indicates that the overall variability within the RR time series is reduced in diabetes. An interesting outcome of this study was that the most significant decrease in HRV in T2DM patients happened within 5–10 years after diagnosis, after which HRV seem to have reached a plateau. This finding is in line with an earlier study where frequency of autonomic neuropathy increased sharply after 5 years of follow-up in T2DM patients (28). The observed plateau phase in HRV after 10 years of follow-up requires further study as it has a bearing on long-term therapy of type 2 diabetic patients and the relevance of intensive insulin therapy.

Medication (anti-hypertensive, antidepressant, and anti-cholesterol medications) did not influence significantly the correlation results given in Table 2. After adjustment for medication, HRV correlations with BGL were only slightly weaker (Mean RR: *r* = −0.221, *p* = 0.002; pNN50: *r* = −0.215, *p* = 0.003; HRVi: *r* = −0.228, *p* = 0.002). This was explained by the positive correlation between BGL and use of anti-cholesterol medication (*r* = 0.145, *p* = 0.046), because of which HRV results between the diabetic patients averaged out when medication was included. HRV correlations with HbA1c or disease duration did not change significantly when computed with correction for medication.

Total cholesterol, LDL, and HDL values were all lower and triglyceride levels were higher for the diabetic patients compared to healthy controls. The different levels of cholesterol are partly explained by the diabetic group using more commonly anti-cholesterol medication compared to control subjects. Triglyceride level was associated with BGL, but not with HbA1c or disease duration. In addition, LDL was negatively correlated with disease duration. The correlation between HbA1c and triglycerides also needs further research with opposing findings being reported in the literature but is a function of diabetes control and medication use as much as of age or diabetes duration (29). Moderate increases in BGL above 5.5 mmol/L have been shown to lead to changes in the redox state and cholesterol as well as triglyceride levels and therefore atherosclerosis (30). HRV has previously been shown by our group to be correlated with the Framingham risk score for CVD disease, which includes presence of diabetes and cholesterol level as risk factors and is directly correlated to the TC/HDL ratio (31).

Although influence of age on HRV has been reported (32, 33), the influence of diabetes duration has received less attention (34). The current research indicates that HRV changes observed in association with duration of diabetes are additive to any effects of age and occur more prominently within the first 5 years of diabetes (35). RMSSD and HF power showed a significant association with duration of diabetes up to 5–10 years indicating that parasympathetic modulation is lost during this period, whereas LF power was reduced significantly in the 10- to 15-year duration group compared to less duration of diabetes, indicating loss of sympathetic influence. These findings corroborate early findings by Ewing and others who suggested that parasympathetic withdrawal precedes sympathetic dysfunction in the modulation of HR (20, 36, 37).

With respect to HbA1c and BGL, a Japanese study has shown that optimal HbA1c and BGL levels to delay microvascular complications in a 8-year follow-up study were HbA1c <6.5% (48 mmol/mol) and BGL <6.1 mmol/L (38). Recent findings of the United Kingdom Prospective Diabetes Study (UKPDS), Diabetes Complications and Control Trial (DCCT), and Australian National Health and Medical Research Council (NHMRC) guidelines recommend a cut-off at 7%. However, this may depend on type of diabetes, duration of diabetes, and comorbidities present (39, 40). Our study indicates that a BGL below the American Diabetes Society cut-off has no effect on HRV changes. However, there are significant effects once BGL rises above 5.6 mmol/L in HRV measures indicating that lower BGL levels have beneficial effects on cardiac health. Similarly, the greatest effect of increased HbA1c occurred above a HbA1c value of 5.7% (39 mmol/mol), which is lower than recommended by previous research but may indicate that cardiac health is more sensitive to minor increases in HbA1c compared to microvascular disease found in the retina or kidneys.

## 5 Conclusion

In summary, our findings indicate that elevated glycemic values have an unfavorable effect on cardiac autonomic function and this effect is pronounced in long-term T2DM patients. Hyperglycemia was associated with moderate increase in mean heart rate and decrease in HRV, whereas duration of diabetes was strongly associated with decrease in HRV. The most significant decrease in HRV related to diabetes was observed to take place within the first 5–10 years of the disease.

## 6 Author Contributions

Mika P. Tarvainen conducted data analyses, interpreted data and wrote the first draft of the manuscript. Tomi Petteri Laitinen contributed to data interpretation and reviewed/edited the manuscript. Jukka Antero Lipponen contributed to data analyses and reviewed/edited the manuscript. David J. Cornforth contributed to data analyses and reviewed/edited the manuscript. Herbert F. Jelinek collected the data, contributed to data analyses and interpretation and reviewed/edited the manuscript.

## Conflict of Interest Statement

The authors declare that the research was conducted in the absence of any commercial or financial relationships that could be construed as a potential conflict of interest.
